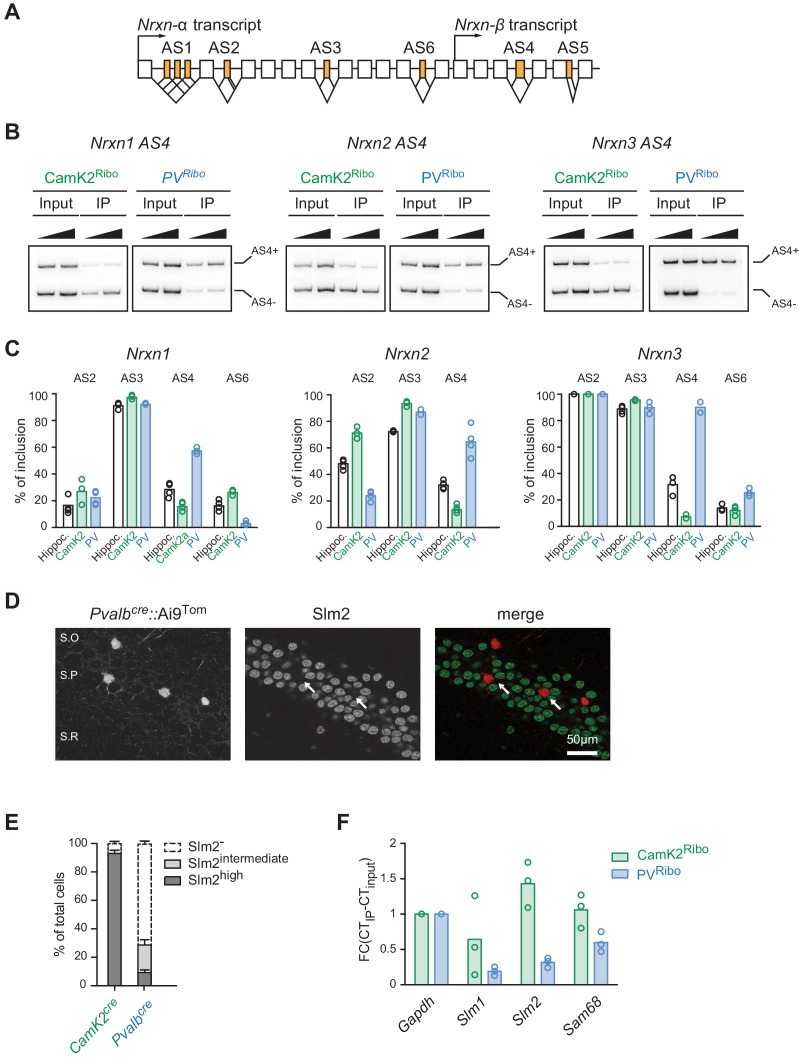# Correction: An alternative splicing switch shapes neurexin repertoires in principal neurons versus interneurons in the mouse hippocampus

**DOI:** 10.7554/eLife.28013

**Published:** 2017-04-25

**Authors:** Thi-Minh Nguyen, Dietmar Schreiner, Le Xiao, Lisa Traunmüller, Caroline Bornmann, Peter Scheiffele

Nguyen T-M, Schreiner D, Xiao L, Traunmüller L, Bornmann C, Scheiffele P. 2016. An alternative splicing switch shapes neurexin repertoires in principal neurons versus interneurons in the mouse hippocampus. *eLife* **5**:e22757. doi: 10.7554/eLife.22757.

Published 13, December 2016

We have become aware of an error in one of the figures in our manuscript. One of the 6 panels in Figure 2B which show radioactive PCR reactions assessing alternative splice insertions in *Nrxn1* was mistakenly taken from reactions performed for *Nrxn2*.

The experiments for *Nrxn1, 2,* and *3* were always performed in parallel and the relative distributions of *Nrxn1* and *2* splice variants detected with this method were very similar. We have replaced the mistaken panel to reflect the accurate data and the article has been corrected accordingly.

The corrected Figure 2 us shown here:

**Figure 1:**
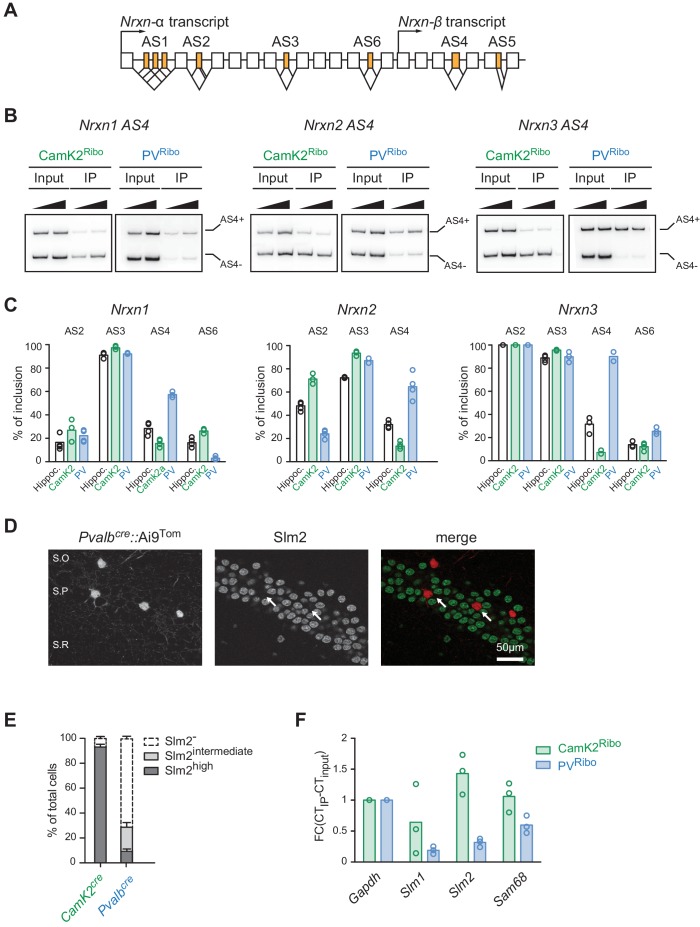

The originally published Figure 2 is also shown for reference:

**Figure 2:**